# Transcriptional and biochemical biomarker responses in a freshwater mussel (*Anodonta anatina*) under environmentally relevant Cu exposure

**DOI:** 10.1007/s11356-020-07660-4

**Published:** 2020-01-13

**Authors:** Gustaf Magnus Oskar Ekelund Ugge, Annie Jonsson, Björn Olsson, Robert Sjöback, Olof Berglund

**Affiliations:** 1 Department of Biology, Lund University, Sölvegatan 37, 223 62 Lund, Sweden; 2 School of Bioscience, University of Skövde, Högskolevägen 3, 541 28 Skövde, Sweden; 3 TATAA Biocenter, Odinsgatan 28, 411 03 Gothenburg, Sweden

**Keywords:** Bivalve, Gene expression, Response variability, Sex effects, Effect size, RT-qPCR

## Abstract

Molecular biomarkers, like gene transcripts or enzyme activities, are potentially powerful tools for early warning assessment of pollution. However, a thorough understanding of response and baseline variation is required to distinguish actual effects from pollution. Here, we assess the freshwater mussel *Anodonta anatina* as a biomarker model species for freshwater ecosystems, by testing responses of six transcriptional (*cat*, *gst*, *hsp70*, *hsp90*, *mt*, and *sod*) and two biochemical (AChE and GST) biomarkers to environmentally relevant Cu water concentrations. Mussels (*n =* 20), collected from a stream free from point source pollution, were exposed in the laboratory, for 96 h, to Cu treatments (< 0.2 μg/L, 0.77 ± 0.87 μg/L, and 6.3 ± 5.4 μg/L). Gills and digestive glands were extracted and analyzed for transcriptional and biochemical responses. Biological and statistical effect sizes from Cu treatments were in general small (mean log_2_ fold-change ≤ 0.80 and Cohen’s *f* ≤ 0.69, respectively), and no significant treatment effects were observed. In contrast, four out of eight biomarkers (*cat*, *gst*, *hsp70*, and GST) showed a significant sex:tissue interaction, and additionally one (*sod*) showed significant overall effects from sex. Specifically, three markers in gills (*cat*, *mt*, GST) and one in digestive gland (AChE) displayed significant sex differences, independent of treatment. Results suggest that sex or tissue effects might obscure low-magnitude biomarker responses and potential early warnings. Thus, variation in biomarker baselines and response patterns needs to be further addressed for the future use of *A. anatina* as a biomarker model species.

**Electronic supplementary material:**

The online version of this article (10.1007/s11356-020-07660-4) contains supplementary material, which is available to authorized users.

## Introduction

High-resolution quantification of early molecular responses to environmental stress is recently made possible by rapid advances within omics technology. Transcriptomics can potentially be used to find biomarkers for pollution; however, gene expression is challenging to relate to whole-organism, population, or ecosystem effects. In contrast, standardized single endpoint ecotoxicity tests, such as mortality and inhibition of growth rate or reproduction (e.g., Organisation for Economic Co-operation and Development 1992, 2004, 2006, 2012), give useful insight into toxic potency of pollutants, but without giving early warnings (Connon et al. 2012). Ideally, molecular responses would be extrapolated to predict effects on higher biological levels. The link is however complicated by a lacking understanding of how baseline gene expression patterns vary with, for example, species, sexes, developmental stages, and seasons (Bahamonde et al. 2016; Fent and Sumpter 2011). Although not always addressed, understanding the variation is thus necessary to discern actual responses from the background noise, for meaningful interpretation of transcriptional data and for successful integration into molecular biomarker panels (Bahamonde et al. 2016). In this study, we have taken initial steps to address baseline and response variation of transcriptional and biochemical biomarkers within and between tissues, sexes, and treatments of a potential model species, the duck mussel (*Anodonta anatina*, family Unionoida).

In ecological monitoring, bivalves are commonly used as bioindicators of aquatic pollution, and various species have been used as model organisms for transcriptional biomarkers in laboratory and field studies (Bigot et al. 2011; Gonzales-Rey et al. 2014; Jaumot et al. 2015; Liu et al. 2014, 2016; Navarro et al. 2011). *Mytilus* sp. is a frequent bivalve model in marine monitoring, and the zebra mussel (*Dreissena polymorpha*) has been proposed as a potential freshwater counterpart (Binelli et al. 2015). However, the zebra mussel is invasive to many Scandinavian and European freshwater systems, which makes us suggest a naturally occurring species, *A. anatina*, as a safer and more ecologically relevant model candidate for field and laboratory studies. This species is widely distributed in Sweden as well as across large parts of Europe (Lopes-Lima 2014). Research on *A. anatina* has to date mostly focused on its phylogeny, morphology, reproduction, and seasonal behavior (Aldridge 1999; Jonsson et al. 2013; Lurman et al. 2014). *A. anatina* and other unionid mussels undergo a complex lifecycle, where gravid females brood eggs and larvae (glochidia) in their gills until parasitic glochidia are released to mature in the gills of host fish (Aldridge 1999; Barnhart et al. 2008; Hinzmann et al. 2013). *A. anatina* has already been used as a model in ecotoxicological field and laboratory studies (Bielen et al. 2016; Falfushynska et al. 2013, 2014; Hartmann et al. 2016; Nugroho and Frank 2011, 2012a, b; Oliviera et al. 2015; Santini et al. 2011), but to our knowledge, transcriptional biomarkers have not previously been studied in the species.

As a primary step to test the potential of *A. anatina* as a model species for molecular biomarkers, we quantified six transcriptional biomarkers after chemical stress. The genes were catalase (*cat*), glutathione-S-transferase (*gst*), heat shock proteins 70 and 90 (*hsp70* and *hsp90*, respectively), metallothionein (*mt*), and superoxide dismutase (*sod*). Catalase and superoxide dismutase are enzymes involved in cellular defense against reactive oxygen species (Bigot et al. 2011; Boukadida et al. 2017), whereas metallothionein plays a main role in metal homeostasis (Fabisiak et al. 1999). Heat shock proteins are involved in general cellular stress response (Liu et al. 2014, 2016) and glutathione-S-transferase in pollutant detoxification (Canesi et al. 1999). As a complement to the transcriptional biomarkers, enzymatic activity of glutathione-S-transferase (GST) was measured in addition to its transcription. Finally, enzymatic activity of acetylcholinesterase (AChE), an enzyme crucial to regulation of nerve signaling (Bocquené and Galgani 1998), was also assessed.

In order to elicit stress responses, Cu was chosen as our experimental model toxicant. In pristine freshwater systems, Cu concentrations are often in the nanogram per liter range (Álvarez-Vázquez et al. 2017; Sander et al. 2013; Vukosav et al. 2014), while in urbanized and polluted areas, in the microgram per liter range (Álvarez-Vázquez et al. 2017; Bhuiyan et al. 2015; Wilson and McMahon 1981), approaching the milligram per liter range in heavily polluted areas (Bhuiyan et al. 2015). According to Swedish environmental quality standards, an annual mean concentration of ≤ 0.5 μg bioavailable Cu/L is required for a “good” status classification (Havs- och vattenmyndigheten 2018). In the current experiment, responses of wild-caught mussels were assessed after exposure to an environmentally relevant range of sublethal Cu levels (additions of 1–100 μg/L) in the laboratory. The a priori hypotheses were that (1) biomarkers respond within the range of Cu concentrations (different relative expressions or activities as compared to the control treatment) and (2) relative response magnitudes differ between treatments and between tissues (gill and digestive gland). In addition to treatment and tissue, mussel sex was included in the model when it became apparent that gravidness affected certain biomarkers.

## Material and methods

### Biomarkers

Six transcriptional (*cat*, *gst*, *hsp70*, *hsp90*, *mt*, *sod*) and two biochemical (AChE, GST) responses were analyzed by reverse transcription quantitative polymerase chain reaction (qPCR) and enzymatic activity assays, respectively. Stress gene selection was based on biomarkers previously used to assess chemical stress in laboratory and field studies on bivalves (Bigot et al. 2011; Gonzales-Rey et al. 2014; Jaumot et al. 2015; Liu et al. 2014, 2016; Navarro et al. 2011). *AlleleID* software (Premier Biosoft, USA) was used for primer design, based on homologous sequences found using the NCBI nucleotide search function (Table A.1). For each gene used, sequences were found for a minimum of one unionid mussel species (order Unionoida) and a minimum of four bivalve species in total. Actual sequences from *Anodonta anatina* were only found and used for primer design for the 28S rRNA gene. The two biochemical markers, AChE and GST, have both been previously used in mussel gills and digestive glands (Lehtonen et al. 2016).

### Mussel collection and maintenance

On the 13th of October 2017, 20 mussels (species *Anodonta anatina*, 83 ± 13 mm shell length) were collected in Vinne å (southern Sweden, 56° 06′ 45″ N, 13° 54′ 35″ E), a freshwater stream with no known point source pollution. Before the start of experiments, the mussels were acclimatized to laboratory conditions for 14 days. During this period, mussels were kept in a 60-L glass aquarium, with 30 L reconstituted freshwater (International organization for standardization 2012), hereafter referred to as freshwater medium, used as medium. The freshwater medium was prepared from distilled water with additions of 294 mg/L CaCl_2_·2H_2_O, 123.3 mg/L MgSO_4_·7H_2_O, 64.8 mg/L NaHCO_3_, and 5.8 mg/L KCl (laboratory reagent grade, Scharlau) and had a nominal hardness of 250 mg/L CaCO_3_. A 5-cm siliceous sand layer was added as bottom substrate to the aquarium. The sand (“Blästersand,” batch 07/17, purchased from Brogård Sand AB, Sweden) originated from Lake Vättern, Sweden, and had a grain size of 0.2–0.7 mm. Before use, the sand was thoroughly washed by hand, by repeatedly stirring it under tap water until the runoff water was clear, and subsequently rinsed with distilled water before added to the aquarium. During acclimatization, the medium was continuously aerated, and three times weekly, 10–20 L medium was renewed. At each time of medium renewal, mussels were fed by additions of *Pseudokirchneriella subcapitata* corresponding to approximately 8 × 10^5^ cells mussel^−1^ day^−1^, except for 48 h before the start of the experiments, during which mussels were starved. During acclimatization, water temperature ranged between 16 and 20 °C, and the light cycle was 16-h light:8-h dark.

### Experimental treatments

A Cu stock solution was prepared from CuCl_2_·2H_2_O (laboratory reagent grade, Fisher Scientific) diluted in freshwater medium to a nominal concentration of 100 mg/L. Stock solution was diluted with freshwater medium in preparation of the three exposure media, to nominal Cu water concentrations of 1, 10, and 100 μg/L, respectively. Freshwater medium without Cu was used for control treatments. One hundred micrograms per liter was selected as the highest concentration in order not to impact filtration and, thus, Cu uptake, based on a previous preliminary Cu exposure experiment with identical setup. In the pre-experiment, prolonged shell closure was observed at additions of 200 μg Cu/L. Glass aquaria with 4.5 L continuously aerated exposure medium and a 5-cm sand layer, prepared as previously described, were prepared approximately 48 h prior to the experimental start. Water (unfiltered) for determination of total Cu concentration was sampled in experimental aquaria at the experiment start, as well as in Vinne å at five time points during December 2017 to December 2018. In addition, the Cu stock solution was sampled. Samples were frozen and subsequently acidified with 1% (v/v) HNO_3_ before Cu determination. Total Cu concentrations in water samples were measured by ICP-MS (*Aurora Elite*, Bruker Daltonics, Germany) for experimental treatments and environmental samples, and by ICP-OES (*Optima 8300*, Perkin Elmer, USA) for the stock solution, respectively. Measured Cu concentrations were < 0.2 μg/L both in the control treatment and after 1 μg/L addition, and on average 0.77 ± 0.87 μg/L and 6.3 ± 5.4 μg/L after addition of 10 μg/L and 100 μg/L, respectively (Table A.2). Estimated Cu sand/water partition coefficients (*K*_*d*_) were 84 and 110 L/kg for the 10 and 100 μg Cu/L additions, respectively (Table A.2). The Cu concentration of the stock solution was 66 mg/L, and sampled background levels in Vinne å ranged between 0.080 and 0.71 μg Cu/L over time (Table A.2).

Mussels were treated in individual aquaria and fed daily with *P. subcapitata* solution (approximately 8 × 10^5^ cells day^−1^). During the experiment, medium temperature was 15 ± 1 °C and the light cycle was 16-h light:8-h dark. After 48 h, 1.5-L medium was renewed in each aquarium, and after 96 h, the exposure was interrupted and mussels were immediately dissected. Gills and digestive glands were extracted and snap frozen in liquid nitrogen. Tissues were stored at − 80 °C until RNA extraction and again until cytosol extraction for biochemical assays. The extractions were made from frozen tissue, and samples were not allowed to thaw in between. During dissection, gravid mussels (hereafter referred to simply as females) were distinguished visually by the presence of eggs or glochidia in the gills (Aldridge 1999; Hinzmann et al. 2013). Male:female sex distribution across treatments was 1:4, 2:3, 3:2, and 3:2 in control, 1, 10, and 100 μg Cu/L, respectively. Upon snap freezing, one female gill sample was lost from each treatment group, i.e., *n =* 4 per treatment remaining for gills.

After biomarker analyses, Cu concentration in remaining tissue samples (16 gill and 14 digestive gland samples, respectively) was analyzed by ICP-SFMS (*Element XR*, Thermo Scientific, Germany) according to Engström et al. (2004). Bioconcentration factors (BCFs) were estimated for each tissue sample where corresponding water concentration was determined (> LOQ).

$$ BCF=\frac{\mathrm{Tissue}\ \mathrm{concentration}\ \left(\upmu \mathrm{g}/\mathrm{kg}\ \mathrm{WW}\right)}{\mathrm{Water}\ \mathrm{concentration}\ \left(\upmu \mathrm{g}/\mathrm{L}\right)} $$

### Gene expression

RNA was extracted from each sample, followed by reverse transcription synthesis of cDNA, which was in turned used in qPCR assays. RNA was extracted from gill and digestive gland samples by using the *SurePrep™ TrueTotal™ RNA Purification Kit* (Fisher Scientific, USA). The A260/A280 ratio was checked using a *NanoVue™ Plus* (GE Healthcare, USA) and was 1.9–2.1 in extracted samples. RNA integrity was assessed qualitatively by gel electrophoresis on 1% agarose gels, and a subset of samples underwent RNA quality assessment using *Fragment Analyzer* (Advanced Analytical, Austria). Based on qualitative and quantitative integrity assessments, all samples were assumed to have an RQN (RNA quality number) of ≥ 6.

For each sample, 1 μg of RNA was converted to cDNA by reverse transcription, using the *Verso cDNA Synthesis Kit* (Thermo Scientific, USA), with random hexamer primers and a reaction cycle of 42 °C for 60 min, 95 °C for 2 min, and 4 °C for 2 min. qPCR assays were performed using *TATAA SYBR® GrandMaster® Mix* (TATAA Biocenter, Sweden), and 400 nM of the respective primer pair, on a *CFX384™ Real-Time PCR Detection System* (Bio-Rad, USA). The reaction program consisted of polymerase activation at 95 °C for 60 s, then 45 cycles of denaturation (95 °C for 5 s), annealing (58 °C for 30 s), and extension (72 °C for 10 s). A dissociation curve (from 60° to 95° C) finalized the program. Primers were purchased from Integrated DNA Technologies (Belgium).

Assay efficiencies were estimated from dilution series of pooled samples. Efficiency determination was performed on a *StepOnePlus™* (Applied Biosystems, USA), using *Maxima SYBR Green/ROX qPCR Master Mix* (Thermo Scientific, USA) for the reactions. Estimated efficiencies were 96–102% (Table A.1), and a 100% efficiency was assumed for all assays. Relative gene expression was determined by the 2^−ΔΔCt^ method (Livak and Schmittgen 2001), normalizing expression by the mean expression of control samples of gill and digestive gland tissue, respectively. The mean of two reference genes, β-actin and 28S rRNA, was used for within-sample normalization.

### Enzyme activity

AChE and GST activity assays were modified from Bocquené and Galgani (1998) and Habig et al. (1974), respectively. Tissue samples were mechanically homogenized on ice in 5:1 (v:w) 0.02 M Na_2_HPO_4_/KH_2_PO_4_ buffer (0.1% Triton-X, pH 7.4) for AChE assays and in 4:1 (v:w) 0.1 M KH_2_PO_4_ buffer (pH 7.4) for GST assays. Following homogenization, samples were centrifuged at 10,000*g* (4 °C, 20 min), and supernatants were stored at − 80° until analysis. Activities were measured for spectrophotometrically at 412 nm for 5 min and 350 nm for 2 min, for AChE and GST respectively. Assays were performed in 96-well microplates (Nunc, Denmark), and absorbance measured using a *SpectraMax 190* plate reader (Molecular Devices, USA). AChE activity was expressed as the hydrolysis rate of acetylthiocholine (Bocquené and Galgani 1998), whereas GST activity was expressed as the rate of glutathione conjugation to 1-chloro-2,4-dinitrobenzene (Habig et al. 1974). Finally, enzymatic activities were normalized by the protein concentration from each extracted tissue sample, as determined by the Bradford (1976) method, using a bovine serum albumin standard curve.

### Statistics

Statistical analyses were run and figures were generated in R version 3.5.2 (R Core team 2018). Measured tissue concentrations were compared between treatment groups by ANOVA. Concentration dependence of tissue Cu concentrations was tested by correlation (Pearson) to measured Cu concentration in the exposure medium. Both concentrations were log_10_-transformed, and samples corresponding to water levels below LOQ were excluded. Gene expressions and enzyme activities were normalized relative to the mean of the respective tissue in the control group and then log_2_-transformed. Transformed values are hereafter referred to as responses and were visualized by principal component analysis, using the R package “factoextra” (Kassambara and Mundt 2017). Responses were then analyzed in a linear mixed model, using the R package “lme4” (Bates et al. 2015). Response was used as the dependent variable for each marker, and full models included the fixed effect terms treatment, sex, and tissue, as well as their interactions (Table A.3). Mussel ID was used as a random effect. Model selection was performed by sequential type I ANOVA analysis, where insignificant effect factors were removed one at a time until remaining factors and/or interactions were significant (*p* < 0.05). For biomarkers where the mixed model selection resulted in a singular fit, the tissues were instead analyzed by separate linear models. Residual normality for biomarker responses and tissue Cu concentrations was assessed by Shapiro-Wilk normality tests and *Q*-*Q* plots. Significant differences in the final models were identified with a Tukey HSD post hoc test, using the “emmeans” package (Lenth 2018). Finally, the R package “simr” (Green and MacLeod 2016) was used for power analysis by simulation in the linear mixed models, while the packages “*pwr*” (Champely 2018) and “sjstats” (Lüdecke 2018) were used for power analysis and effect size assessment of ANOVAs.

## Results

**Fig. 1:**
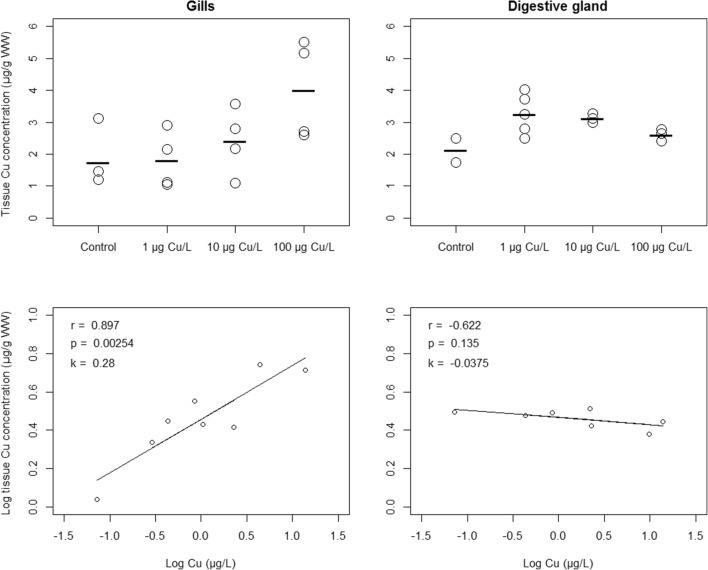
Cu concentration (μg/g WW) in gill (upper left) and digestive gland samples (upper right), and their respective correlation with measured water concentration (below). For tissue sample Cu analysis, 16 gill and 14 digestive gland samples remained after transcriptional and biochemical analyses. Circles represent single samples and black bars show group means

Treatment groups did not differ significantly in measured tissue concentration in either gills or digestive glands (*p* > 0.05), despite gills demonstrating an approximately twofold higher mean concentration in the 100 μg/L group as compared to control (Fig. 1). Yet, the gill Cu concentrations were significantly correlated with measured water Cu concentrations (Fig. 1). In contrast, digestive gland Cu concentrations were not correlated with exposure levels (Fig. 1). Across exposures in which water Cu was > LOQ, median BCF was 3400 L/kg WW and 1500 L/kg WW in gills and digestive gland, respectively.

**Fig. 2:**
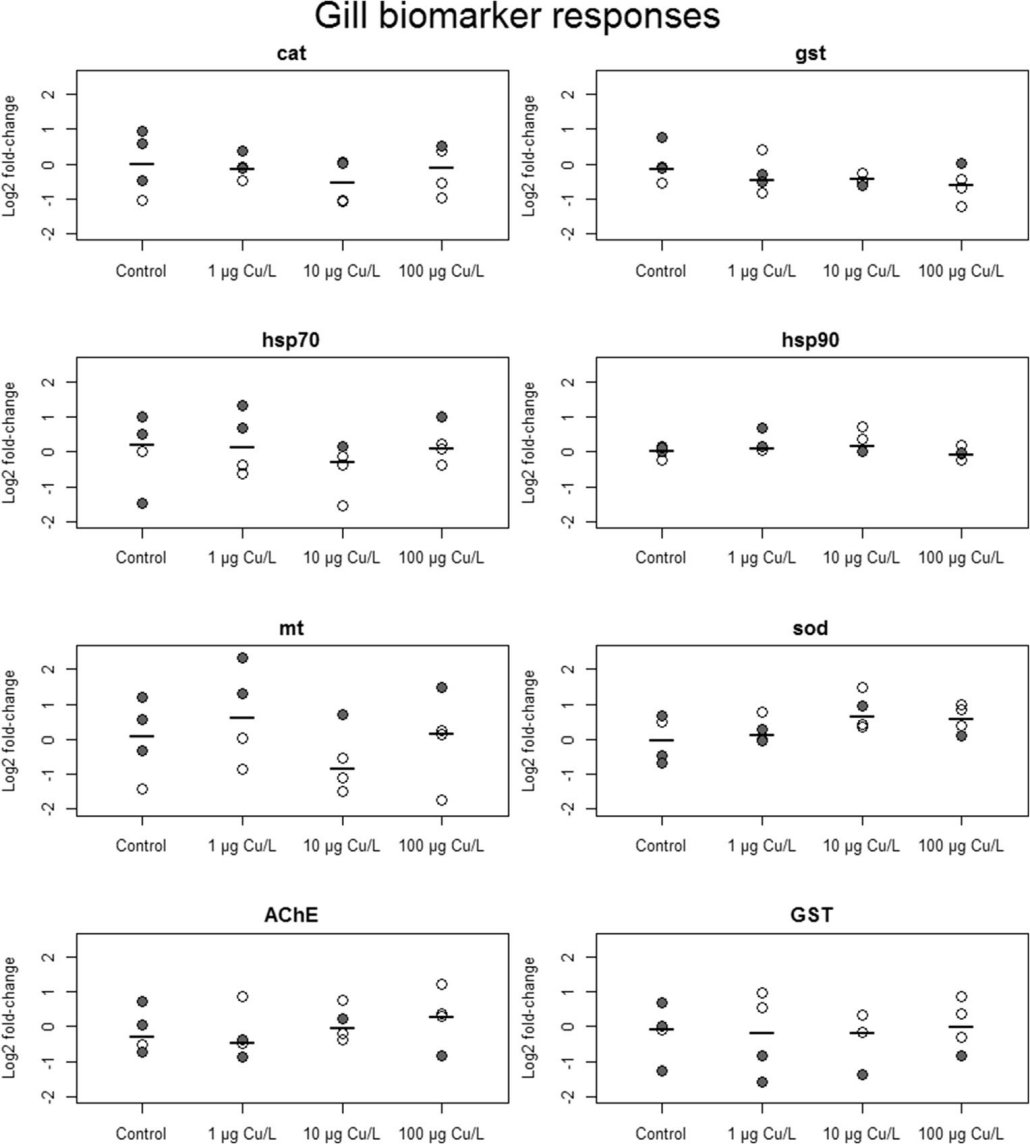
Biomarker responses (log_2_ fold-change relative control treatment) by copper treatment, in gill tissue (*n* = 4 per treatment) from *A. anatina*. Gray and white points correspond to gravid and non-gravid mussels, respectively, while black bars show treatment median responses

**Fig. 3:**
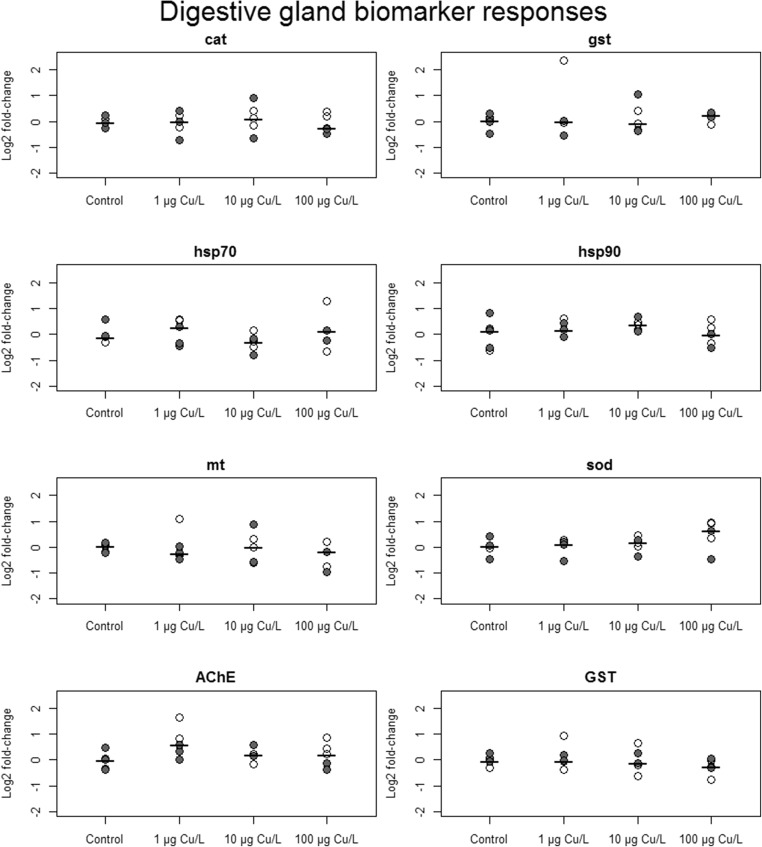
Biomarker responses (log_2_ fold-change relative control treatment) by copper treatment, in digestive gland tissue (*n* = 5 per treatment) from *A. anatina*. Gray and white points correspond to gravid and non-gravid mussels, respectively, and black bars show treatment median responses

**Fig. 4:**
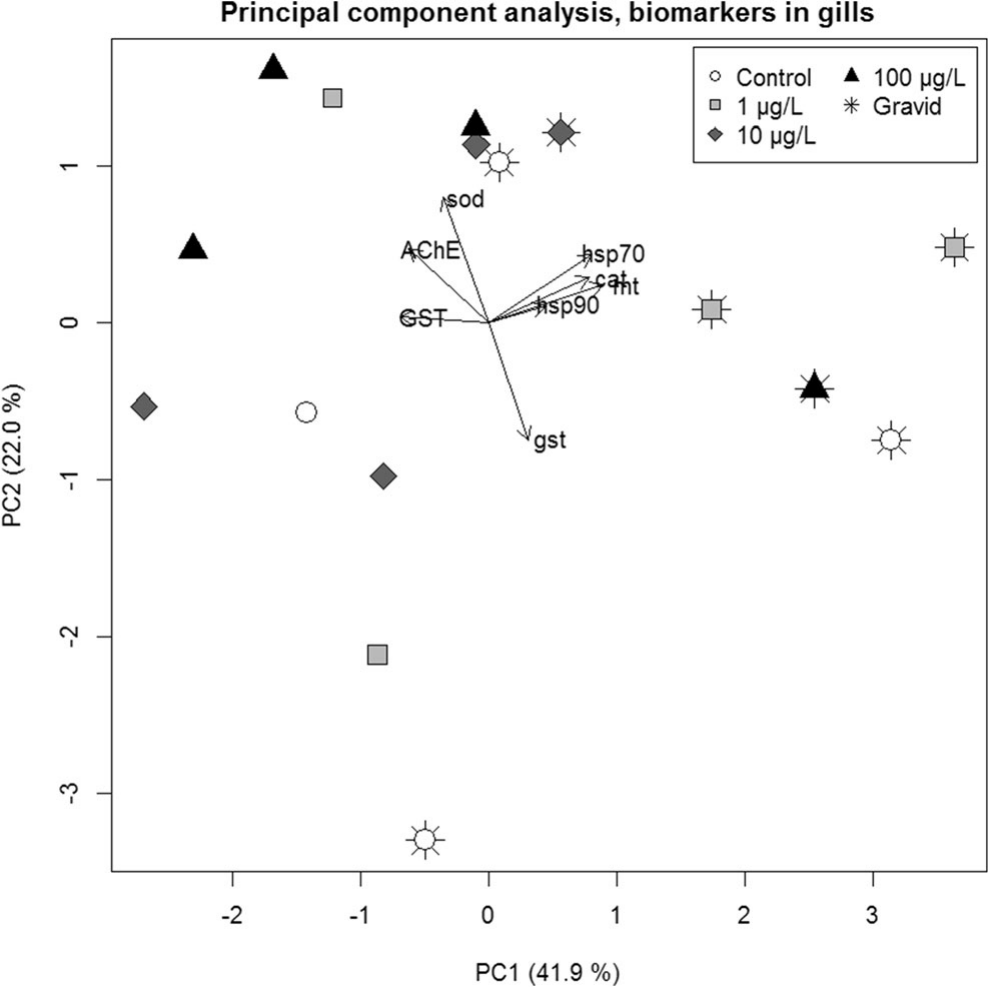
Principal component analysis of eight molecular biomarker responses in gills of *A. anatina* (*n* = 16). Arrows imply the contribution of the respective biomarkers to PC1 and PC2

**Fig. 5:**
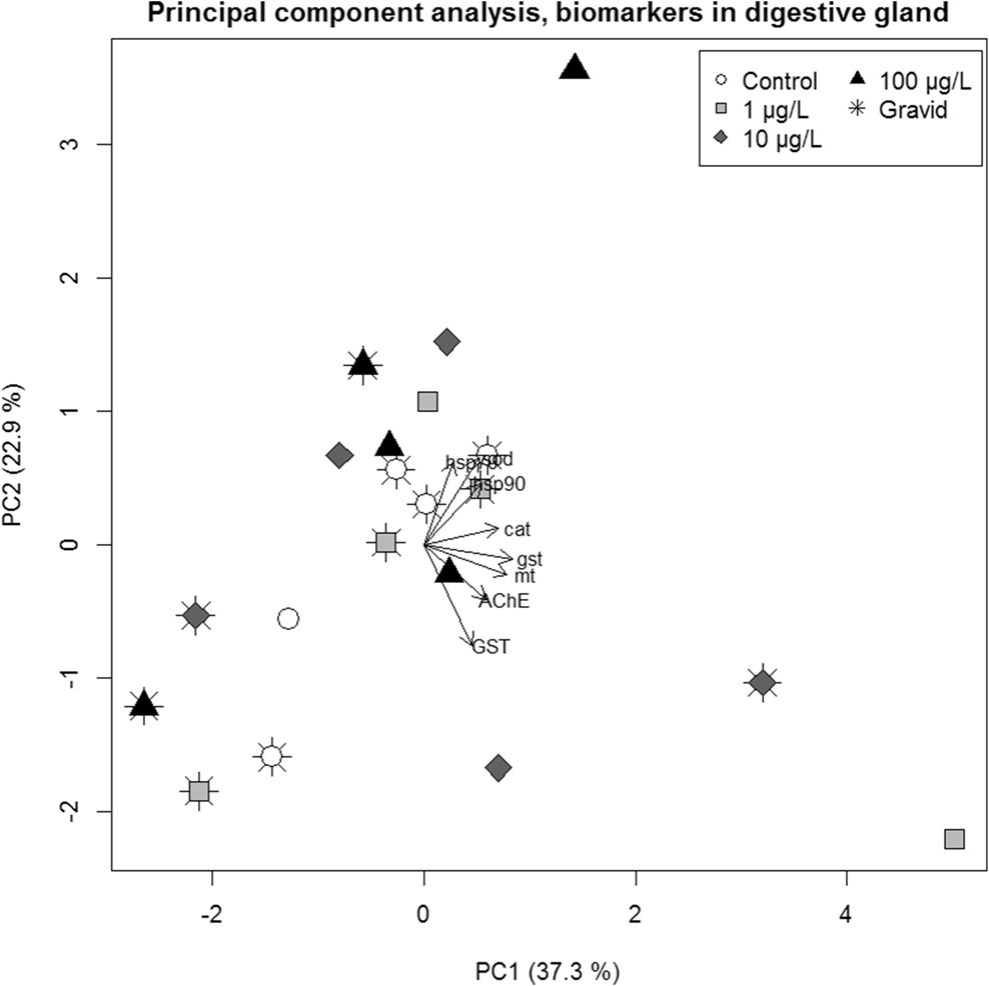
Principal component analysis of eight molecular biomarker responses in digestive gland of *A. anatina* (*n* = 20). Arrows imply the contribution of the respective biomarkers to PC1 and PC2

Overall, sex and tissue effects and/or interactions were prominent for all biomarkers except *hsp90*. No distinct responses from Cu treatments were demonstrated. Biomarker responses showed overlapping distributions in all experimental treatments, and no biomarker showed discernable differences between treatment groups, neither in gills nor in digestive glands (Figs. 2 and 3). Gill responses generally showed higher variation for all markers except *gst* and *hsp90*. Standard deviations of *mt*, AChE, and GST were consistently greater in gills than in digestive glands across treatments (Table A.4). For *mt* and GST, overall standard deviation in gills was more than twice the size of that in digestive glands. Similarly, no treatment effects could be inferred from principal component analysis of biomarker responses separated by tissue (Figs. 4 and 5). In gills however, there was a separation of females and males along PC1, with only little overlap (Fig. 4), implying a sex difference in expressions of most notably *cat*, *mt*, and *hsp70* (Table A.5).

### Linear (mixed) models

In the full models, observed power was low for treatment effects (< 0.4) and treatment interactions (< 0.6) (Table A.3). Treatment effects were insignificant in all markers (*p* > 0.05), and only AChE showed an initially significant treatment interaction (Table A.3). Due to insignificance in a subsequent step of the AChE model selection, the treatment term was however excluded in all final models (Table 1). For *mt* and AChE, the final models were linear models separated by tissue, whereas the other markers were analyzed by linear mixed models. In *cat*, *gst*, GST, and *hsp70*, there was a significant sex:tissue interaction, whereas *sod* expression only showed significant sex effects (Table 1). Significant sex effects were also observed in gill *mt* expression and digestive gland AChE activity. In contrast, for *hsp90* expression, as well as *mt* expression in digestive gland and AChE activity in gills, there were no significant effects (Table 1). Males showed a significantly higher *sod* expression than females in general and a higher GST and AChE activity in gills and digestive gland, respectively. Females on the other hand displayed a significantly higher expression of *mt* in gills, as well as a higher gill-specific expression of *cat* compared to males (Table 1). In addition, mean *hsp70* expression was higher in females than males, and the linear mixed model implied a sex:tissue interaction that was however not significantly confirmed post hoc (Table 1). Males displayed significantly higher relative *cat* and *gst* expressions in digestive gland as compared to gills, while females showed significantly higher GST activity in digestive gland as compared to gills (Table 1).

**Table 1:** Effects from sex, tissue, and their interactions in the final (mixed) linear model for analyzed biomarkers, as well as the observed power for the effects (based on 100 simulations)

Biomarker	Final model	(Fixed) effect	Effect size (Δlog_2_ as compared to control)	Obs. power (1 − *β*)	Model term sign. level	Observed differences (post hoc)
*cat*	Response ~ Sex*Tissue + (1|ID)	Intercept (F:Dg)	− 0.077	–	–	Sex differences: Dg:F < M (*p* = 0.84) *G:F* > *M* (*p = 0.0087*) Tissue differences: F:Dg < G (*p* = 0.44) *M:Dg* > *G* (*p = 0.045*)
		Sex (M:Dg)	0.17	0.32	*p* = 0.089	
		Tissue (F:G)	0.34	0.22	*p* = 0.32	
		Sex:tissue (M:G)	− 0.96	*0.86*	*p = 0.0061*	
*gst*	Response ~ Sex*Tissue + (1|ID)	Intercept (F:Dg)	0.060	–	**–**	Sex differences: Dg:F < M (*p* = 0.73) G:F > M (*p* = 0.31) Tissue differences: *Dg* > *G* (*p = 0.0024*) F:Dg > G (*p* = 0.99) *M:Dg > G* (*p = 0.0009*)
		Sex (M:Dg)	0.26	0.04	*p* = 0.62	
		Tissue (F:G)	− 0.060	*0.94*	*p = 0.0020*	
		Sex:tissue (M:G)	− 0.74	*0.82*	*p = 0.0067*	
*hsp70*	Response ~ Sex*Tissue + (1|ID)	Intercept (F:Dg)	− 0.11	–	**–**	Sex differences: Dg:F < M (*p* = 0.86) G:F > M (*p* = 0.067) Tissue differences: F:Dg < G (*p* = 0.25) M:Dg > G (*p* = 0.39)
		Sex (M:Dg)	0.21	0.12	*p* = 0.28	
		Tissue (F:G)	0.56	0.05	*p* = 0.84	
		Sex:tissue (M:G)	− 1.0	0.79	*p = 0.021*	
*hsp90*	Response ~ (1|ID)	Intercept	0.15	–	**–**	**–**
*mt*	Gill response ~ Sex +1	Intercept (F)	1.0	**–**	**–**	Sex difference: *F* > *M* (*p = 0.00051*)
		Sex (M)	− 1.8	*0.99*	*p = 0.00051*	
	Dig. gland response ~ 1	Intercept	− 0.086	**–**	**–**	–
*sod*	Response ~ Sex + (1|ID)	Intercept (F)	0.054	–	–	Sex difference: *F* < *M* (*p = 0.012*)
		Sex (M)	0.44	0.75	*p = 0.011*	
AChE	Gill response ~ 1	Intercept	0.0083	–	**–**	**–**
	Dig. gland response ~ Sex +1	Intercept (F)	0.088	–	**–**	Sex difference: *F* < *M* (*p = 0.049*)
		Sex (M)	0.43	0.56	*p = 0.049*	
GST	Response ~ Sex*Tissue + (1|ID)	Intercept (F:Dg)	0.022	–	–	Sex differences: Dg:F > M (*p* = 0.95) *G:F* < *M* (*p = 0.0031*) Tissue differences: *F:Dg* > *G* (*p = 0.025*) M:Dg < G (*p* = 0.39)
		Sex (M:Dg)	− 0.13	0.49	*p* = 0.066	
		Tissue (F:G)	− 0.76	0.19	*p* = 0.29	
		Sex:tissue (M:G)	1.1	*0.89*	*p = 0.0028*	

Significance level is presented for all model terms, and differences were tested post hoc for significant model terms (*p* < 0.05). Italicized entries imply observed powers ≥ 0.8 and *p* values < 0.05. *Dg* digestive gland, *G* gills, *F* females, *M* males

### Treatment effects

Treatment effects were further analyzed in a simplified model of tissue-separated treatment effects, completely disregarding sex effects. One-way ANOVA analyses for each marker, separated by tissue, showed that no treatment response differed significantly from control. Observed power ranged from 0.097 to 0.55 (Table A.6), and the largest observed mean response magnitudes for each marker ranged between a log_2_ fold-change of 0.26–0.80 and 0.13–0.69, in gills and digestive gland, respectively (Table A.6). For certain markers (*hsp90* in gills, *sod* in gills and digestive gland, AChE in digestive gland), an approximate doubling of sample size would have given a power of 0.8 with current effect size, while others (e.g., *cat* and *gst* in digestive gland, GST in gills) would require more than a tenfold increase in sample size to achieve the requested power (Table A.6).

## Discussion

### Cu exposure and uptake

Bioavailability and potential uptake of Cu depends on various water parameters, and toxicity decreases with, for example, dissolved organic carbon (DOC) and water hardness (Arnold et al. 2009; Gillis et al. 2008, 2010; Wang et al. 2009; Giacomin et al. 2013). Importantly, bioavailability depends on Cu partitioning, which in turn is affected by both water parameters and sediment organic content (European Copper Institute 2008). For sandy sediments, Hassan et al. (1996) demonstrated Cu partition coefficients (*K*_*d*_) ranging from 0.6 to 149 L/kg, in line with estimated *K*_*d*_ values from our experiment. Under current settings, the substrate is therefore assumed to be an important factor to reduce Cu bioavailability and potential stress responses, by adsorbing a major fraction of the added Cu.

Different patterns of Cu uptake and tissue distribution have been reported for various bivalve species and exposure conditions (Canesi et al. 1999; García-Navarro et al. 2017; Nugroho and Frank 2011; Sakellari et al. 2013; Serafim and Bebianno 2009; Won et al. 2016). Whole-body BCFs of 3300 L/kg WW (Potipat et al. 2015) and 576 to approximately 15,000 L/kg DW (Le et al. 2011; Rosioru et al. 2016) have been proposed. In gills specifically, a BCF of 42 L/kg WW was modeled for *Corbicula fluminea* (Chen et al. 2010). For aquatic organisms in general, McGeer et al. (2003) reported a mean whole-body BCF of 1200 ± 1800 L/kg WW under the exposure range of 1–10 μg Cu/L. Considering the variation in reported Cu uptake, our observed tissue concentrations and BCFs are well within the expected range.

Under current settings, the gill Cu levels were positively correlated with water concentrations, implying Cu uptake under the highest exposures. Although treatment groups did not significantly differ in gill Cu concentrations, this is likely due to variation in actual exposure within and between groups. In contrast, digestive glands showed no correlation, suggesting that observed Cu concentrations rather reflect the tissue baseline. Potential, not necessarily mutually exclusive, explanations to differences between in gills and digestive glands include different accumulation and/ or elimination rates. Gills constitute a first-line defense against harmful toxicants, and gill uptake might be of importance for bivalve regulation of reactive oxygen species (ROS) under Cu stress (Won et al. 2016). However, fecal elimination, via the digestive gland, has been proposed as a major route for metal elimination in *A. anatina* (Nugroho and Frank 2011). At low exposure concentrations, an efficient Cu elimination might thus result in no net uptake to digestive glands. Also, accumulation rates may differ between tissues (Canesi et al. 1999; Serafim and Bebianno 2009). Thus, the current exposure period and/or Cu concentrations were potentially not enough for digestive gland net uptake to occur.

### Biomarker responses

Experimental Cu exposures were in the lower range as compared to previous studies on bivalve transcriptional responses, and response magnitudes in our experiment were generally lower in both tissues than previously reported. All mean responses (converted back from the log_2_ scale) were within the range of 0.67–1.7 relative to control. With regard to the biochemical markers, *Mytilus galloprovincialis* exposed for 96 h to 5 or 15 μg Cu/L demonstrated an approximate 1.5-fold increase in GST activity (Perić and Burić 2019). Similar to our results, AChE activity was however unaffected except in a binary exposure of Cu and chlorpyrifos (Perić and Burić 2019). In contrast, in transcriptional markers, the freshwater mussel *Corbicula fluminea* displayed *mt*, *cat*, *sod*, and *gst* responses varying between a tenfold decrease to a fourfold increase in gills and digestive gland after acute (12 h) exposure to a nominal concentration of 10 μg Cu/L (Bigot et al. 2011). Our largest observed mean responses approximately correspond to acute *C. fluminea* response magnitudes at the nominal concentration of 50 μg Cu/L (Bigot et al. 2011), however not regarding the same genes and tissues. Even larger response magnitudes have been demonstrated in, for example, larvae and hemocytes of *Mytilus* spp. after various Cu stress exposures. After exposure to 10–20 μg Cu/L, significant upregulations ranging between two- and eightfold increases have been demonstrated across all our assessed transcriptional markers (Boukadida et al. 2017; Liu et al. 2014, 2016). Without a treatment to serve as positive control, we currently cannot be certain of what level of Cu stress would be required to induce corresponding response magnitudes in *A. anatina*.

### Sex and tissue differences

Except for AChE activity, digestive glands did not demonstrate sex-specific differences. Considering that no uptake was demonstrated for this tissue, for most of the tested biomarkers, there appears to be little sex influence on baseline signal. In contrast, gill signals differed distinctly in four markers, with females showing higher *cat*, *mt*, and *hsp70* (non-significant) expressions and lower GST activity than males. In *A. anatina*, gravidness develops sequentially from early summer until glochidia are released in late winter/early spring (Hinzmann et al. 2013). Since mussels were sampled in October, observed differences in gills could potentially be explained by gravidness, by the eggs and glochidia directly interfering with biomarker signals, and/or by protective molecular mechanisms in the gravid mussel gill. Acute toxicity (48 h LC_50_) to *A. anatina* glochidia has been demonstrated at 18.9 μg Cu/L (Kováts et al. 2010) and at 6.5–32 μg Cu/L in glochidia of various other unionid species (Wang et al. 2007). This range only overlaps the highest measured Cu concentrations of our experiment, whereas our mussels were exposed for a longer period. Still, we observed no treatment:sex:tissue interaction, suggesting that observed differences were mainly due to gravidness-induced baseline variation rather than responses to Cu exposure. Regardless of mechanistic explanation, relative sex differences in gills may possibly change over the course of the season depending on female gravidness.

In addition to tissue-specific sex differences, some markers showed a sex-specific difference in biomarker signal between tissues (*cat*, *gst*, GST). However, although Cu uptake patterns differed, no treatment:tissue or treatment:sex:tissue interaction was observed, i.e., no tissue difference in actual stress response can be concluded. In the absence of such interactions, remaining tissue differences rather reflect overall variation within and between sexes.

### Tested biomarkers

The currently tested biomarkers represent a subset of general stress responses and have all previously been shown to respond to Cu exposure in bivalves (Bigot et al. 2011; Boukadida et al. 2017; Goswami et al. 2014; Liu et al. 2014, 2016; Perić et al. 2017). Apart from metallothionein, which is cytoprotective by binding to metals (Fabisiak et al. 1999), the biomarkers were however not expected to respond specifically to Cu exposure. Rather, responses would mainly reflect mechanisms of cellular protection from, for example, oxidative stress (Bigot et al. 2011; Boukadida et al. 2017) or protein damage (Liu et al. 2014, 2016). The primary objective was to identify biomarkers responding to general chemical stress in *A. anatina*, rather than to respond specifically to Cu exposure. Since we failed to detect stress responses at current elevated Cu concentrations, additional markers, specific to Cu or other target pollutants, might be needed before *A. anatina* can be successfully used in, for example, environmental monitoring.

An ideal biomarker for early warnings of chemical stress should be one that gives a strong response at levels below, or time points before, responses translate to whole-organism effects. Despite low exposure concentrations, Cu in our experiment was approaching water levels at which we previously observed prolonged shell closure in preliminary experiments (unpublished). That is, further concentration increases would risk higher-level effects by affecting mussel behavior. In practice, the window for detecting early warnings might thus be quite narrow for acute Cu stress, further increasing the need of a strong biomarker response.

In our experiment, response magnitudes were generally small, but an approximate doubling of the sample size would have substantially increased the power for *hsp90*, *sod*, and AChE in one or both tissues. However, most of the markers would require drastically increased sample sizes to improve power. Ideally, large effects should be obtained biologically by high response magnitude and/or statistically by low natural variation, even at small sample sizes. At current Cu stress, *A. anatina* background variation however obscures potential low-magnitude responses, and the tested markers appear to require quite drastically increased sample sizes for detection of early warnings. Thus, no successful *A. anatina* biomarker candidate can be singled out, and further search for and/or evaluation of biomarkers is necessary Alternatively, successful assessment of low-level stress may ultimately depend on response pattern analysis of multiple stress biomarkers.

### Conclusion

Either larger response magnitudes or substantially larger sample sizes would have been required to quantify molecular stress responses under the current Cu concentrations. Our results, specifically displayed by sex differences in gills, illustrate how potential low-magnitude stress responses, and potentially early warnings, might be obscured by variations in baseline biomarker expression/activity. However, for successful development of environmentally relevant biomarker models, natural variation should preferably be addressed rather than avoided. Being abundant, sessile, and ecologically relevant, we suggest further studies on *Anodonta anatina* as a biomarker model species. A better understanding of response magnitudes, variation, and links to higher biological levels is needed to realize the potential of *A. anatina* as a freshwater biomarker model.

## Electronic supplementary material

ESM 1 (PDF 169 kb).
