# Supplementary material for: Transcriptional and biochemical biomarker responses in a freshwater mussel (Anodonta anatina) under environmentally relevant Cu exposure
Source: Environ Sci Pollut Res Int. 2020 Jan 13;27(9):9999–10010. doi: 10.1007/s11356-020-07660-4 (PMC7089896; doi:10.1007/s11356-020-07660-4)
Supplement: Supplementary file 1 — (PDF 169 kb). [file 11356_2020_7660_MOESM1_ESM.pdf]

Appendix

Transcriptional and biochemical biomarker responses in a freshwater mussel (*Anodonta anatina*) under environmentally relevant Cu exposure

Gustaf M.O. Ekelund Ugge[a,b†], Annie Jonsson[b], Björn Olsson[b], Robert Sjöback[c], Olof Berglund[a]

[a]Department of Biology, Lund University, Sölvegatan 37, 223 62 Lund, Sweden

[b]School of Bioscience, University of Skövde, Högskolevägen 3, 541 28 Skövde, Sweden

[c]TATAA Biocenter, Odinsgatan 28, 411 03 Gothenburg, Sweden

[†]Corresponding author at: Department of Biology, Lund University, Sölvegatan 37, 223 62 Lund, Sweden.
E-mail address: gustaf.ekelund_ugge@biol.lu.se

Table A.1. Primer and assay details for qPCR assays of *Anodonta anatina* stress gene expression. Primer sequences, efficiency, standard curve slope, intercept and R^2. Also presented is the estimated amplicon length, and accession numbers for gene sequences used for primer design. fwd = forward primer, rev = reverse primer.

Gene	Sequence (5’-3’)	Efficiency (%)	Slope	Intercept	R^2	Amplicon size (bp)	Sequence references for primer design (accession number)
β-actin (*β-act*)	fwd: CAAACTGGGATGATATGGA<br>rev: CATCTTTTCTCTGTTGGC	101	-3.32	23.5	0.894	128	KT923181.1, HM045420.1, AF082863.1, AF172606.1, AF157491.1
28S rRNA (28S)	fwd: ATCCTTGCTCGTCACGAC<br>rev: GTACCAACCCTTCCTACG	98.5	-3.37	13.1	0.999	75	KX822588.1, KC703865.1, MF414393.1, MF414392.1, MF414391.1, MF414390.1, KX822589.1, KX822630.1, U82350.1
Catalase (*cat*)	fwd: GGAAGACTGACCAGGGTAT<br>rev: CCTCAGCGATGGCATTGTA	96.0	-3.43	21.5	0.997	108	HQ148707.1, KU363383.1, FJ608579.1, EU145723.1, HM188565.1, EU407490.1
Glutathione-S-transferase (*gst*)	fwd: GTCCAACACCATGCTGAG<br>rev: GTAGTCCTCCACTCCATCAT	99.2	-3.35	21.5	0.998	106	HQ166721.1, AY885666.1, EU145724.1, EF194203.1, AY557404.1, AJ557140.1
Heat shock protein 70 (*hsp70*)	fwd: GGTATTGAGACGGCTGGT<br>rev: CACACCAGGCTGGTTGTC	102	-3.30	21.8	0.997	111	KX758099.1, HQ148706.1, KT923183.1, AF172607.1, EF526096.1, AB122063.1, KJ123764.1
Heat shock protein 90 (*hsp90*)	fwd: TACCATTGCCAAATCTGG<br>rev: ACACCAAACTGCCCAATCA	101	-3.31	24.8	0.995	84	HQ180224.1, GU433881.1, EF687776.1, KR633143.1
Metallothionein (*mt*)	fwd: ATGCAACTGCCTTGAGAC<br>rev: ACTTTACATCCAGGACACTT	100	-3.32	22.5	0.997	114	EF185127.1, GQ184290.1, KJ019820.1, KJ019821.1, U67347.1, AJ577130.1, AJ577131.1, AJ243263.1
Superoxide dismutase (*sod*)	fwd: GCTACGGTCATTCCACTCT<br>rev: CCAGTTATCTCACCAGTTATGTTC	97.4	-3.39	24.9	0.995	171	FJ194441.1, KU363382.1, FJ608580.1, KT724303.1, EU145730.1, AY377970.2

Table A.2. Concentrations of total Cu in sampled water of experimental treatments, Cu stock solution and environmental samples. The limit of quantification (LOQ) was 0.2 µg/L.

Treatment	Samples (n)	Mean Cu concentration (g/L)<br>(min – max)	Estimated K_d[a] (L/kg)<br>(min – max)
Control	3	<LOQ	-
1 µg Cu/L	3	<LOQ	-
10 µg Cu/L	5	$7.7 * 10^{-7}$<br>($7.2 * 10^{-8}$ – $2.2 * 10^{-6}$)	84<br>(22 – 1 000)
100 µg Cu/L	5	$6.3 * 10^{-6}$<br>($1.0 * 10^{-6}$ – $1.4 * 10^{-5}$)	110<br>(42 – 690)
100 mg Cu/L (stock solution)	1	$6.6 * 10^{-2}$	-
Background (environmental samples)	5[b]	$3.9 * 10^{-7}$<br>($8.0 * 10^{-9}$ –$7.1 * 10^{-7}$)	-

[a] K_d was estimated by the formula $K_d = C_s/(C_w/S.C.)$ (Hassan *et al*. 1996). C_w is the measured water concentration (µg/L) and S.C. is the sand to medium ratio (kg/L) in the aquarium. C_s is the concentration (µg/kg) assumed to adsorb to the sand, and was calculated as $C_s = (66\ 000$ µg/L*V_{stock} – 5 L*C_w)/1.5 kg. 66 000 µg/L was the measured Cu concentration of the stock solution, and V_{stock} is the volume (L) of stock solution added to the respective treatments. Each aquarium contained 5 L water and approximately 1.5 kg of sand.

[b] Background levels were sampled every three months over the course of a year, in December 2017 and March, June, September and December 2018.

Table A.3. Full linear mixed models for investigated biomarkers. Observed power (based on 100 simulations) and significance level are presented for model fixed effect terms.

Biomarker	Full model	Fixed effect	Obs. power (1 – β)	Significance level (p)
cat	Response ~ Treatment*Sex*Tissue + (1|ID)	Treatment	0.02	0.93
		Sex	0.20	0.17
		Tissue	0.14	0.39
		Treatment:Sex	0.06	0.73
		Treatment:Tissue	0.19	0.58
		Sex:Tissue	0.64	0.019
		Treatment:Sex:Tissue	0.12	0.74
gst	Response ~ Treatment*Sex*Tissue + (1|ID)	Treatment	0.05	0.92
		Sex	0.04	0.74
		Tissue	**0.92**	0.0026
		Treatment:Sex	0.21	0.34
		Treatment:Tissue	0.50	0.099
		Sex:Tissue	0.79	0.016
		Treatment:Sex:Tissue	0.25	0.32
hsp70	Response ~ Treatment*Sex*Tissue + (1|ID)	Treatment	0.23	0.27
		Sex	0.11	0.39
		Tissue	0.07	0.83
		Treatment:Sex	0.03	1.0
		Treatment:Tissue	0.08	0.92
		Sex:Tissue	0.78	0.017
		Treatment:Sex:Tissue	0.49	0.21
hsp90	Response ~ Treatment*Sex*Tissue + (1|ID)	Treatment	0.30	0.12
		Sex	0.05	0.79
		Tissue	0.08	0.81
		Treatment:Sex	0.26	0.20
		Treatment:Tissue	0.07	0.98
		Sex:Tissue	0.06	0.79
		Treatment:Sex:Tissue	0.27	0.38
mt	Response ~ Treatment*Sex*Tissue + (1|ID)	Treatment	0.21	0.26
		Sex	0.77	0.0053
		Tissue	0.18	0.37
		Treatment:Sex	0.12	0.82
		Treatment:Tissue	0.39	0.25
		Sex:Tissue	**1.0**	0.00027
		Treatment:Sex:Tissue	0.19	0.73
sod	Response ~ Treatment*Sex*Tissue + (1|ID)	Treatment	0.37	0.22
		Sex	0.31	0.14
		Tissue	0.43	0.062
		Treatment:Sex	0.07	0.88
		Treatment:Tissue	0.28	0.25
		Sex:Tissue	0.12	0.72
		Treatment:Sex:Tissue	0.14	0.50
AChE	Response ~ Treatment*Sex*Tissue + (1|ID)	Treatment	0.14	0.44
		Sex	0.54	0.038
		Tissue	0.47	0.066
		Treatment:Sex	0.52	0.045
		Treatment:Tissue	0.41	0.19
		Sex:Tissue	0.06	0.99
		Treatment:Sex:Tissue	0.14	0.66
GST	Response ~ Treatment*Sex*Tissue + (1|ID)	Treatment	0.07	0.96
		Sex	0.53	0.039
		Tissue	0.18	0.30
		Treatment:Sex	0.27	0.27
		Treatment:Tissue	0.23	0.50
		Sex:Tissue	**0.90**	0.0041
		Treatment:Sex:Tissue	0.25	0.70

Table A.4. Biomarker response standard deviations in gills and digestive glands, by treatment and overall across treatments.

Biomarker	Gills (*n*=4 per treatment)					Digestive gland (*n*=5 per treatment)				
	Control	1 µg Cu/L	10 µg Cu/L	100 µg Cu/L	Overall	Control	1 µg Cu/L	10 µg Cu/L	100 µg Cu/L	Overall
cat	0.918	0.346	0.628	0.713	0.642	0.184	0.433	0.583	0.359	0.390
gst	0.547	0.527	0.178	0.516	0.472	0.296	1.14	0.592	0.192	0.625
hsp70	1.08	0.912	0.747	0.579	0.818	0.337	0.488	0.356	0.717	0.495
hsp90	0.177	0.286	0.346	0.175	0.272	0.590	0.267	0.224	0.448	0.409
mt	1.14	1.40	0.959	1.33	1.19	0.179	0.632	0.624	0.473	0.498
sod	0.675	0.364	0.527	0.415	0.554	0.314	0.332	0.300	0.578	0.411
AChE	0.644	0.741	0.498	0.836	0.646	0.343	0.611	0.309	0.484	0.494
GST	0.808	1.19	0.727	0.755	0.807	0.213	0.487	0.489	0.314	0.389

Table A.5. Biomarker weights in principal components 1 and 2, in *A. anatina* gills and digestive gland, respectively.

Biomarker	Gills		Digestive gland	
	PC1	PC2	PC1	PC2
cat	0.430	0.215	0.406	0.0947
gst	0.166	-0.565	0.482	-0.0805
hsp70	0.437	0.325	0.160	0.458
hsp90	0.243	0.0764	0.311	0.338
mt	0.491	0.182	0.455	-0.164
sod	-0.194	0.602	0.300	0.482
AChE	-0.342	0.356	0.339	-0.303
GST	-0.376	0.0276	0.262	-0.556

Table A.6. Observed mean responses and power analysis of copper effects in gills and digestive glands of *A. anatina*. Required sample size is rounded off to integers to give a power of approximately 0.8.

Biomarker	Gills (*n*=4 per treatment)				Digestive gland (*n*=5 per treatment)			
	Largest obs. mean response (Δlog_2)	Treatment effect size (Cohen's f)	Obs. power (1-β)	Req. Δn (1-β≈0.8)	Largest obs. mean response (Δlog_2)	Treatment effect size (Cohen's f)	Obs. power (1-β)	Req. Δn (1-β≈0.8)
cat	-0.51 (10 µg/L)	0.33	0.13	+23 (575%)	0.13 (10 µg/L)	0.22	0.099	+53 (1 060%)
gst	-0.58 (100 µg/L)	0.52	0.29	+7 (175%)	0.37 (1 µg/L)	0.21	0.097	+55 (1 100%)
hsp70	-0.48 (10 µg/L)	0.40	0.18	+14 (350%)	-0.32 (10 µg/L)	0.42	0.25	+12 (240%)
hsp90	0.27 (10 µg/L)	0.64	0.42	+4 (100%)	0.36 (10 µg/L)	0.43	0.27	+11 (220%)
mt	0.69 (1 µg/L)	0.43	0.21	+11 (275%)	-0.37 (100 µg/L)	0.36	0.19	+17 (340%)
sod	0.80 (10 µg/L)	0.69	0.48	+3 (75%)	0.47 (100 µg/L)	0.52	0.38	+6 (120%)
AChE	0.26 (100 µg/L)	0.30	0.12	+26 (650%)	0.69 (1 µg/L)	0.64	0.55	+3 (60%)
GST	-0.33 (10 µg/L)	0.17	0.071	+90 (2 250%)	-0.26 (100 µg/L)	0.40	0.23	+13 (260%)
